# A Naked Eye Refractive Index Sensor with a Visible Multiple Peak Metamaterial Absorber

**DOI:** 10.3390/s150407454

**Published:** 2015-03-26

**Authors:** Heli Ma, Kun Song, Liang Zhou, Xiaopeng Zhao

**Affiliations:** Smart Materials Laboratory, Department of Applied Physics, Northwestern Polytechnical University, Xi’an 710129, China; E-Mails: marcos12@126.com (H.M.); songkun@mail.nwpu.edu.cn (K.S.); mzlmql@126.com (L.Z.)

**Keywords:** refractive index sensor, naked eyes, metamaterial absorber, bottom-up

## Abstract

We report a naked eye refractive index sensor with a visible metamaterial absorber. The visible metamaterial absorber consisting of a silver dendritic/dielectric/metal structure shows multiple absorption peaks. By incorporating a gain material (rhodamine B) into the dielectric layer, the maximal magnitude of the absorption peak can be improved by about 30%. As the metamaterial absorber is sensitive to the refractive index of glucose solutions, it can function as a sensor that quickly responds to variations of the refractive index of the liquid. Meanwhile, since the response is presented via color changes, it can be clearly observed by the naked eyes. Further experiments have confirmed that the sensor can be used repeatedly.

## 1. Introduction

Metamaterials (MMs) are artificial media exhibiting many extraordinary electromagnetic (EM) properties that do not exist in the naturally available materials, for instance, negative refractive index, super-resolution and trapped rainbow effects [1,2,3]. With these unique properties, MMs may lead to important practical applications [4,5,6,7,8]. As an important branch of MMs, metamaterial absorbers (MAs) are of great current interest due to their intriguing properties including high absorption of EM energy and hyperspectral imaging [9]. In order to maximize the absorption of EM waves, meticulously designed metal resonator/dielectric/metal composite structures are used to control the effective permittivity and permeability of a MA. By optimizing the geometric parameters, the MA can neither reflect nor transmit EM waves at some selective frequency band. The EM energy is therefore weakened to zero with a high absorption coefficient. In the past few years, research on MAs has undergone an almost explosive growth, and considerable progress has been made in this burgeoning field. The current interest in the field is turning to the enticingly potential applications in areas such as sensors, thermal emitters, and photovoltaic devices [10,11]. However, to date the reported previously MAs could only realize near unity absorbance from the microwave to the infrared region [12,13,14,15,16,17]. Thus, how to realize a MA operating in visible light region is still a challenge for researchers.

Recently, much attention has been focused on optimizing MM properties with gain materials [18,19,20]. Xiao *et al.* [18] incorporated a gain material in the high-local-field area of a fishnet MM to improve the loss compensation. This idea provides a flexible route to control the properties of MMs. Fang *et al.* [20] investigated the optical response of a realistic split-ring resonator (SRR) array with a gain layer underneath. Results showed that the SRR resonance was enhanced and the real and imaginary parts of permeability μ became larger than those without gain layers. From these works we can see that the relation between the enhancement of resonance and absorption property of MA deserves careful research.

In this article, we experimentally demonstrate a MA consisting of silver dendritic cells with different diameters that exhibits multiband absorption peaks in the visible light region. By incorporating moderate amounts of rhodamine B (RhB, 99% pure, laser grade) into the dielectric layer, the absorption magnitudes of the MA can be effectively enhanced. Interestingly, the MA can quickly respond to the variations of the concentrations of glucose solutions to yield color changes on the surface of the MA observable by the naked eyes, enabling important application as a medical sensor.

## 2. Experimental

**Figure 1:**
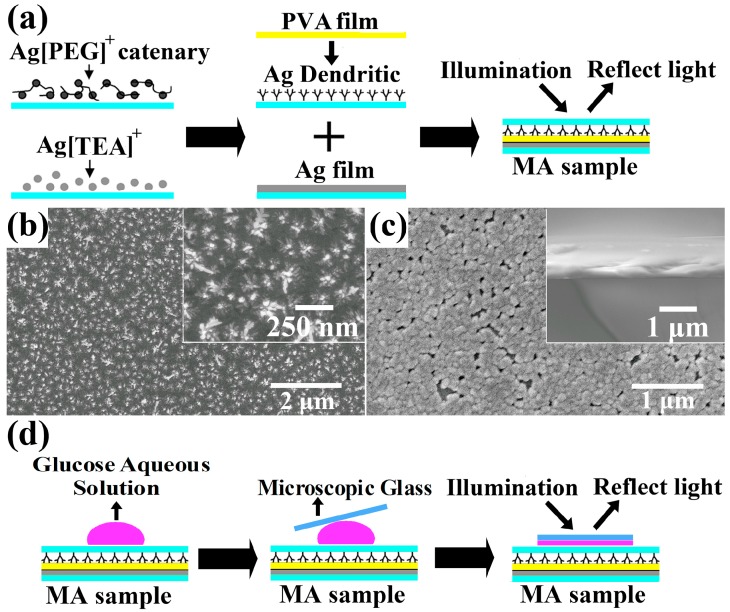
(**a**) The fabrication process of the multiband visible MA; (**b**) Scanning electron microscope (SEM) image of the prepared silver dendritic units—the illustration is an enlarged drawing of silver dendritic units; (**c**) SEM image of the silver metal plane—the inset is the cross-section of the silver metal plane.

The fabrication process of this multiband visible MA obtained by a “bottom-up” electrochemical deposition method is shown in Figure 1a. The preparation of the silver dendritic cells has been reported in detail [21]. We used ITO conductive glass as cathode, a planar and smooth high-purity silver slice as anode. A mixed solution of silver nitrate (AgNO_3_)/polyethylene glycol-20000 (PEG-20000, concentration 9.9%~10.7%) was injected into the interspace of the two electrodes. Under the condition of direct current (DC) voltages of 0.8~0.9 V for 80~100 s at room temperature, silver dendritic cells with different diameters ranging from 70 nm to 280 nm were fabricated, as shown in Figure 1b. The preparation of the silver metal plane used the same electrochemical deposition device. Firstly, 0.1 g of AgNO_3_ was added into 2 mL ultrapure water (UPW) and stirred sufficiently. Then, 1 mL triethanolamine (TEA) was added into the solution drop by drop. Immediately, a brown precipitate formed in the mixture, however, as the amount of added TEA increased further, the precipitate gradually disappeared and the solution became clear. Essentially, the silver ions were complexed with TEA molecules. With the solution of AgNO_3_/triethanolamine (TEA) mixture a silver metal plane with a thickness of about 400 nm was prepared, as shown in Figure 1c. In the experimental procedure, the deposition current decreased with increasing deposition time. The ITO conductive glass coated with silver dendritic cells and silver metal plane took on silver-grey and silver-white colors, respectively. A polyvinyl alcohol (PVA) solution with concentration of 1%~3% was dropped onto the surface of the prepared silver dendritic cells. By this vertical deposition method under ambient conditions, the silver dendritic cells were covered with a PVA dielectric interlayer with a thickness of about 50~100 nm [21]. Finally, the silver dendritic cells/PVA dielectric interlayer and silver metal plane were combined face-to-face to form the MA sample. The sample area finallyobtained is about 30 mm × 12 mm.

In practical terms, two kinds of MA samples were prepared. The silver dendritic cells of the first kind of MA samples were obtained under a deposition voltage of 0.8 V, a deposition time of 80 s, a PEG concentration of 10.7% and 1.0% PVA. The silver dendritic cells of the second kind of MA samples were prepared with a deposition voltage of 0.8 V, a deposition time of 100 s, a 9.9% concentration of PEG and 3.0% PVA. In addition, to investigate the effect of gain material (RhB) on the absorption properties of the MA samples, we incorporated RhB into the PVA dielectric layer instead of a pure PVA layer.

## 3. Results and Discussion

### 3.1. Measurements of Absorption Properties of MA Samples

The MA samples were measured via a UV-visible spectrophotometer (Hitachi UV-4100) in reflection mode in the wavelength region of 500~800 nm. The incident and reflection angles were both 5°, and the reflection spectra were normalized to that of a silver mirror. Since the silver metal plane is dense and its thickness is considerably larger than the penetration depth of light wave, the transmittance can be ignored. As a result, the absorptivity can be calculated as **A** = 1 − **R**, where **A** and **R** refer to the absorptivity and reflectivity of the sample, respectively.

**Figure 2:**
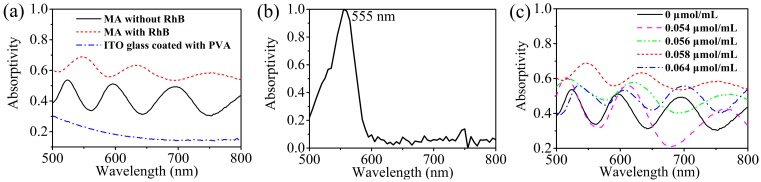
(**a**) The absorption spectra for MA without RhB, MA with RhB, and the ITO glass coated with PVA in the testing region of 500~800 nm; (**b**) The inherent absorption spectrum of the RhB; (**c**) The absorption spectra of the MA evolve with different concentrations of RhB.

Zhu *et al.* [22,23] have investigated the absorption performances of the MAs with periodically and randomly arrayed silver dendritic cells via simulations. The results indicated that the power loss might be attributed to the electric resonance caused by the main trunk of dendritic cells and magnetic resonance induced by the antisymmetrical current between the dendritic cells and metal plane. Figure 2a shows the measured absorption spectra of the proposed MA, MA integrated with RhB, and ITO glass coated with PVA. The MA samples were prepared under the first kind of deposition conditions. It is seen that the present MA with different silver dendritic cells can simultaneously resonate at different wavelengths leading to multiband absorption. However, the quantity and density of the silver dendritic cells with the similar geometric parameters are so few that it is unfavorable for the generation of strong resonance at a certain frequency. Consequently, a unit absorptivity cannot be realized and the maximum absorptivity of the MA is about 0.537 at 524 nm. After incorporating RhB into the PVA dielectric layer, it is of significance that the magnitudes of the absorption peaks of the proposed MA are effectively enhanced. The maximal absorptivity is dynamically increased by about 30%. This result arises due to the inherent absorption of the RhB which results in a significant decrease of the reflectance (Figure 2b). Meanwhile, compared with the sample without RhB, the absorption peaks of the sample integrated with RhB show significant red shifts. These spectral shifts are attributed to the fact that the maximum absorption peak of RhB is at 555 nm wavelength which is in the long-wavelength part of the absorption peak for the MA sample without RhB, so after the inclusion of RhB, the absorption peaks were red shifted [24]. Furthermore, there remains a weak absorption of about 0.14 to 0.3 owing to the inherent absorption of the ITO glass and PVA.

Figure 2c illustrates the evolution of the absorption spectra of the MAs with different concentrations of RhB. The magnitudes and locations of the absorption peaks measured under different concentrations of RhB are shown in Table 1. It is obvious that in response to the increasing concentration of RhB, the magnitudes of the absorption peaks gradually increase and rise to their maximal values (0.692/546 nm, 0.641/632 nm, 0.596/752 nm) under the concentration of 0.058 μmol/mL. However, with the further increase of the concentration of RhB, the magnitudes of the absorption peaks gradually decrease. When the concentration of RhB is 0.064 μmol/mL, the average absorption decreases significantly. This phenomenon may be explained by the fact that the RhB will generate photoluminescence under a direct light illumination, effectively increasing the reflection of the MA sample, which leads to a significant decrease of the final absorption of the MA sample [24].

**Table 1:** The magnitudes and locations of absorption peaks under different concentrations of RhB.

Concentration (μmol/mL)	The Magnitudes/Locations of Absorption Peaks (nm)		
0	0.537/524	0.513/596	0.502/698
0.054	0.608/516	0.564/612	0.430/768
0.056	0.600/518	0.584/620	0.516/770
0.058	0.692/546	0.641/632	0.596/752
0.064	0.560/536	0.538/606	0.506/700

### 3.2. Measurements of the Sensibility of MA to Variations of the Refractive Index for Glucose Aqueous Solutions

**Figure 3:**
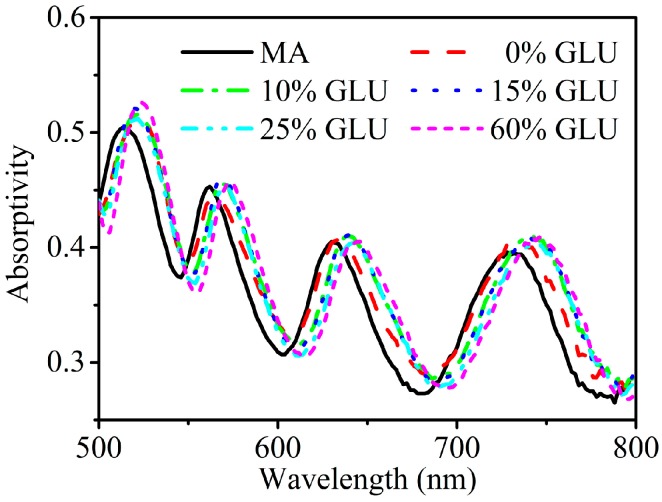
The effect on the absorption spectra for MA with drops of different concentrations of GLU on its surface. The black solid line shows the absorption spectrum of the MA.

To further investigate the intriguing properties of the present MA, we measured the sensibility of MA to the variations of the refractive index of aqueous glucose (GLU) solutions of different concentrations. Generally, refractive index sensors based on photonic crystals which need to inject the tested liquid into the microcavity can be used only once or require a complex process for reuse, extremely limiting their practical applications [25,26]. In our experiments, the GLU aqueous solution drop was overlaid on the surface of ITO glass deposited with silver dendritic cells and pressed into a thin film by a microscopic glass. The fabrication and measurement processes are shown in Figure 1d. In this manner, the PVA layer does not dissolve in the aqueous solution and the sample can be repeatedly used just by removing the GLU aqueous solution on its surface. The MA sample was obtained via the second deposition conditions. We tested five kinds of concentrations for GLU (0, 10, 15, 25, and 60%) with corresponding refractive indexes of about 1.333, 1.348, 1.356, 1.375, and 1.439 at 589 nm, respectively [10,27]. The refractive index change range is only 0.106. As shown in Figure 3, there are four absorption peaks at 514, 562, 630 and 732 nm before any solution is dropped.

After successively dropping 0, 10, 15, 25 and 60% GLU solutions, the frequencies of the four absorption peaks all show slight red shifts, and the maximum red-shift values of the four absorption peaks are 12, 12, 14 and 16 nm, respectively. This fact indicates that the frequencies of the absorption peaks of the MA are sensitive to the variations of refractive index of GLU solutions. Therefore, the MA holds great promise for detecting refractive index changes of a sensing agent. A similar phenomenon has also been confirmed in a previous paper [28]. However, compared with the sensor in [28] which can only accomplish single-frequency responses, our present sensor can produce multiband responses to the refractive index changes. For the four absorption peaks, the average variations of refractive index (Δλ/Δn) are about 113.2, 113.2, 132.1, and 150.9 nm/RIU, which is of the same order as those of previously proposed sensors [10,26]. Meanwhile, the magnitudes of absorption peaks do not diminish evidently with the variations of refractive index.

**Figure 4:**
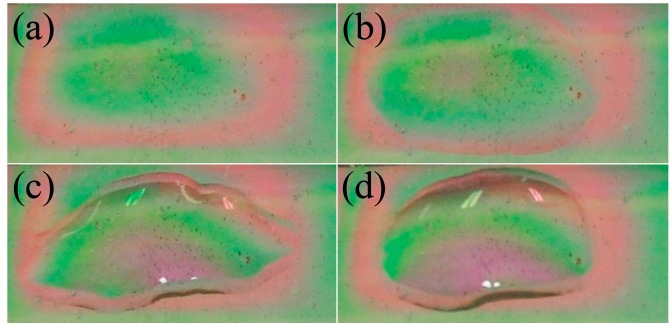
The photographs of the surface of MA with different concentrations of GLU (**a**) without any solution; (**b**) with UPW; (**c**) with 20% GLU solution; (**d**) with 60% GLU solution.

The corresponding color change of the red shift effect mentioned above can be directly observed by the naked eyes. In practical terms, three kinds of GLU aqueous solutions with different concentrations (0%, 20% and 60%) were selected to observe the color changes on the surface of the sample. In Figure 4a, the center of the MA without any solution added is green. As the concentration of the aqueous GLU solution increases, instead of green, a magenta color gradually appears, as shown in Figure 4b–d. Finally, the center is dominated by magenta. The origin of this phenomenon lies in the red shift effects of absorption peaks as well as the color blending of light with different frequencies. Compared with traditional refractive index sensors, a small change of less than 0.05 in the liquid refractive index can be qualitatively distinguished just with the naked eyes. In the meanwhile, the detection process can be completed within a few minutes and the operation is very easy.

## 4. Conclusions

In conclusion, we have fabricated a MA with randomly distributed silver dendritic cells which exhibits multiband absorption in the visible light region for green, yellow and red light, respectively, via a “bottom-up” electrochemical deposition method. Incorporating a moderate gain material in the PVA dielectric layer is beneficial for the enhancement of absorption, and the absorption magnitude can be improved by about 30%. In addition, the absorption peaks of MA are sensitive to variations of the refractive index of glucose solutions. It is shown that the corresponding color changes on the surface of the MA can be clearly observed by the naked eyes. Due to the aforementioned unique properties, the considered MA is extremely promising for applications in medical sensors, solar cells, and visible stealth technology.
